# Determination of the synergistic anti-influenza effect of Huangqin Su tablet and Oseltamivir and investigation of mechanism of the tablet based on gut microbiota and network pharmacology

**DOI:** 10.1186/s12906-023-03858-4

**Published:** 2023-02-04

**Authors:** Xuran Cui, Xibao Liu, Feng Wang, Kun Lou, Junping Hong, Hequn Bai, Rongchu Chen, Yang Yang, Qingquan Liu

**Affiliations:** 1 Beijing Hospital of Traditional Chinese Medicine, Capital Medical University, Beijing, 100010 China; 2 Beijing Institute of Chinese Medicine, Beijing, China; 3 Beijing Key Laboratory of Basic Research With Traditional Chinese Medicine On Infectious Diseases, Beijing, China; 4 CSPC ZhongQi Pharmaceutical Technology Co., Ltd, Shijiazhuang, China

**Keywords:** Influenza, HuangqinSu tablet, Gut microbiota, Network pharmacology

## Abstract

Huangqin Su (HQS) tablet is mainly composed of baicalein which has been evaluated for its ability to inhibit influenza. The present study aimed to investigate the effect of HQS and oseltamivir phosphate (OS) (single or combination therapy) on influenza-induced acute pneumonia in male and female ICR mice. The regulatory effect of HQS on gut microbiota was also studied by using 16 s rDNA sequencing, and the targets and mechanisms of HQS against influenza were comprehensively analyzed by network pharmacology. Pharmacodynamic results, including lung index and pathological changes, showed that HQS exhibited significant anti-influenza efficacy and could improve the efficacy of low-dose OS (*P* < 0.05 and *P* < 0.01, respectively). The results of 16 s rDNA sequencing revealed that HQS modulated the gut microbiota and remarkably enriched the abundance of *Lactobacillus*. The findings of network pharmacology research suggested that the anti-influenza mechanism of HQS was related to TLRs, MAPK, and other signal transduction pathways. Taken together, this study identified the possibility of the combined use of HQS and OS and demonstrated the role of HQS in modulating the gut microbiota of mice against influenza. Network pharmacology studies also suggested that the anti-influenza effect of HQS was related to TLRs, MAPK, TNF, and other signaling pathways.

**Supplementary Information:**

The online version contains supplementary material available at 10.1186/s12906-023-03858-4.

## Introduction

Influenza causes sporadic pandemics and seasonal outbreaks every year, which severely threaten human health and life [1]. According to the World Health Organization, influenza causes more than 3 million severe infections and 290,000 deaths each year [2]. Antiviral drugs and vaccines target the virus itself and are the main treatment methods against influenza [3, 4]. Three main types of antiviral drugs are widely used for the clinical treatment of influenza, namely M2 channel blockers (amantadine and amantadine), neuraminidase inhibitors (oseltamivir [OS]), and RNA polymerase inhibitors (ribavirin); however, these drugs frequently cause adverse reactions and drug resistance in patients [5, 6]. The endonuclease enzyme inhibitor baloxavir is a new drug recently approved by the USA and Japan in 2018; however, its long-term efficacy remains to be studied [7]. Although influenza vaccines are essential in preventing seasonal influenza epidemics, they must be modified regularly to match the constant antigenic drift and antigenic transformation of the influenza virus [8]. Finding new anti-influenza drugs targeting the host from the aspects of inflammatory and immune-related pathways or the use of combination therapy with two or more drugs to improve treatment efficacy could be an important approach in anti-influenza research [9–11].

Huangqin Su (HQS) tablet is mainly composed of baicalein, a monomer compound with a content of more than 90%, which has been evaluated for the anti-influenza effect. Baicalein is a flavonoid compound isolated from the *Scutellaria baicalensis*, an important variety of traditional Chinese medicine [12]. *S. baicalensis* is listed as a traditional Chinese medicinal herb in the Shennong Materia Medica, and its dried roots have been used for a long time to treat lung infections, liver diseases, inflammation, etc. [13, 14]. Presently, *S. baicalensis* is widely used as a medicinal plant in China and many other Asian countries [15, 16]. Baicalein is the only active ingredients of HQS and shows a good inhibitory effect on RNA viruses [17, 18]. Thus, HQS has the potential to treat influenza virus infection; however, it remains unclear whether HQS could be combined with the first-line drug OS to enhance the antiviral efficacy for treating influenza infection. If this approach is feasible, it will open up new possibilities to combat influenza. Thus, studies focused on this aspect are required to gain further knowledge.

Previous studies have shown that gut microorganisms can increase the bioavailability of flavonoids in influenza-infected mice, and baicalein plays an important role in this process [19]. A growing body of evidence suggests that gut microbiota plays an important role in health and disease [20, 21]. As noted previously, following infection with the influenza virus, the number of host pathogenic bacteria such as *Enterococcus* and *Enterobacter* increased, while the number of probiotic bacteria such as *Lactobacillus* and *Lactococcus* decreased [22]. Beneficial modulation of gut microorganisms can significantly improve patient survival and quality of life. Prevention of influenza by modulating gut commensal probiotics such as *Lactobacillus* has been clinically proven to be an effective approach. This approach enhances host immune function and significantly improves flu symptoms [23]. One of the mechanisms of *Lactobacillus* is to stimulate the host to produce type I interferon, which in turn enhances protection against influenza [24]. Therefore, focusing on revealing the probiotics that can regulate the structure of intestinal microorganisms and drive the immune function of the body provides a strong development direction for research on new anti-influenza drugs. Here, we speculate that HQS can target gut microbiota and enhance the immune function of the host to combat influenza.

Baicalein can regulate immunity through different targets and play a role in maintaining human health. For example, baicalein targets GTPase-mediated autophagy to eliminate liver tumor-initiating stem cell-like cells that are resistant to mTORC1 inhibition [25]. It can also use the pathogenic island-1 (SPI-1) type III secretion system (T3SS) effector of *Salmonella typhimurium* (SPI-1) and the translocation enzyme as targets to inhibit bacterial invasion of epithelial cells and exert antibacterial properties [26]. It can also improve learning and memory impairment in SAMP8 mice, which is dependent on the inhibition of A/β and the RAGE/JAK2/STAT1 cascade [27]. Therefore, screening the common targets of baicalein-related targets and influenza virus infection-related targets is an important approach to explore the possible mechanisms of baicalein against influenza virus infection. This could be achieved through network pharmacology, which is a discipline that studies the pathogenesis of diseases by constructing and analyzing biological networks and is particularly suitable for studying multitarget drugs [28–30].

**Fig. 1:**
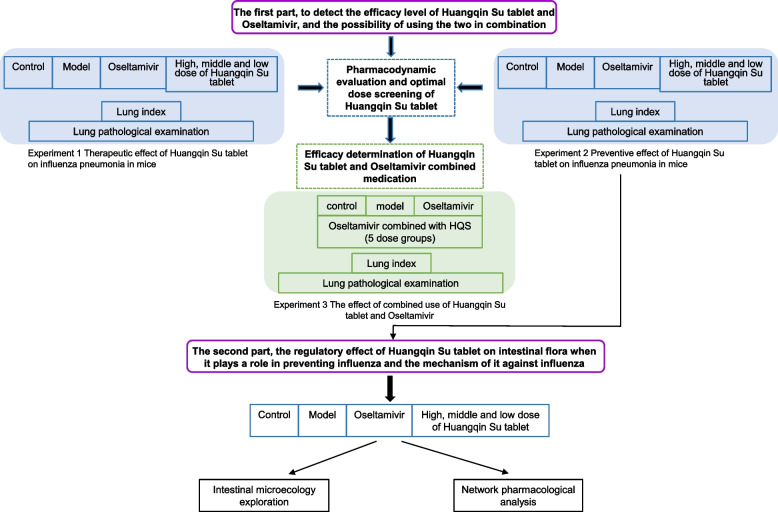
Graphical abstract of the experiment protocol used in this study

In summary, the present study aims to discuss the synergistic anti-influenza effect of HQS and OS and provide a solid scientific background for its application. We also studied the regulatory effect of HQS on the intestinal microbiota of mice during the exertion of its pharmacodynamic effect and analyzed its multitarget regulation mechanism through network pharmacology. The overall structural diagram is shown in Fig. 1.

## Materials and methods

### Drugs and reagents

HQS tablets (baicalein, purity > 98%) were obtained from Shijiazhuang Zhongqi Pharmaceutical Co., Ltd. (Shijiazhuang, China). OS phosphate (Tamiflu) was obtained from Roche Pharma (Schweiz) Ltd. (Shanghai, China). Bio-Plex Pro™ Mouse Cytokine Th1/Th2 Panel 8-Plex was purchased from Bio-Rad.

### Virus

Influenza virus H1N1 (A/FM/1) was supplied by the Institute of Chinese Materia Medica, China Academy of Chinese Medical Sciences (Beijing, China).

### Animals and drug preparation

Specific pathogen-free ICR mice (13–15 g) were obtained from Beijing Vital River Laboratory Animal Technology Co., Ltd. (Beijing, China). The mice were housed in IVC cages and raised at 22 °C ± 1 °C with a relative humidity of 50% ± 10% and a 12-h light/dark cycle. Mice were given free access to feed and water. HQS or OS was dissolved in distilled water for animal experiments. HQS was diluted to final concentrations of 4.12, 8.25, and 16.5 mg/mL. OS was diluted to 1.37 mg/mL. Mice were intragastrically administered HQS (82.5, 165, and 330 mg/kg) or OS (27.5 mg/kg) with a volume of 0.2 mL/10 g of body weight.

### Protocol for animal experiments

The influenza virus-infected mouse model was established as described previously [31], with slight modifications.

#### Experiment 1 Therapeutic effect of HQS on influenza pneumonia in mice

Sixty mice were randomly divided into 6 groups (*n* = 10 per group), namely the control group (distilled water); model group (distilled water); HQS low-, middle-, and high-dose groups (82.5, 165, and 330 mg/kg/d, respectively); and OS group (27.5 mg/kg/d). The mice were anesthetized with isoflurane and intranasally inoculated with 40 µL of the FM1 virus solution (15 LD_50_), except the control group. Mice in the control group were intranasally administered with an equivalent volume of normal saline. One hour after infection, the infected mice in the HQS group were administered low-, middle-, and high-dose HQS, 0.2 mL/10 g body weight, once a day by gavage for 5 days. Mice in the OS group were intragastrically administered OS, while mice in the control and model groups were intragastrically administered distilled water. On day 5 post infection, mice were euthanized by isoflurane overdose and sacrificed for lung index calculation and histopathological evaluation.

#### Experiment 2 Preventive effect of HQS on influenza pneumonia in mice

Sixty mice were randomly divided into a control group (distilled water); model group (distilled water); HQS low-, middle-, and high-dose groups (82.5,165, and 330 mg/kg/d, respectively); and OS group (27.5 mg/kg/d), with 10 mice in each group. Mice in the HQS or OS group were treated with low-, middle-, and high-dose of HQS or OS by gavage once a day for 3 days. Mice in the control and model groups were intragastrically administered distilled water. Following the last administration, mice were anesthetized with isoflurane and intranasally administered 40 µL of the FM1 virus solution (15 LD_50_), except for the control group. Mice in the control group were intranasally administered with 40 µL of normal saline. The mice in all groups were observed every day. On day 4 post infection, the mice were euthanized by isoflurane overdose and sacrificed for lung index calculation, histopathological evaluation, cytokine detection, and gut microbiome analysis.

#### Experiment 3 Effect of combined use of HQS and OS

To assess whether the use of HQS could increase the pharmacodynamic level of OS or reduce the dose of OS, we first analyzed the results of experiments 1 and 2 and concluded that the effect of the middle dose of HQS was better. Next, in experiment 3, the middle dose of HQS and different doses of OS were used in combination to determine the level of efficacy. The experimental method was as follows.

Seventy mice were randomly divided into 7 groups (10 mice per group), namely control group (distilled water); model group (distilled water); OS group; and Co 1 (HQS 165 mg/kg/d with OS 27.5 mg/kg/d), Co 2 (HQS 165 mg/kg/d with OS 14 mg/kg/d), Co 3 (HQS 165 mg/kg/d with OS 7 mg/kg/d), and Co 4 (HQS 82.5 mg/kg/d with OS 14 mg/kg/d) groups. Except for the control group, mice were anesthetized with isoflurane and intranasally administered 40 µL of the FM1 virus solution (15 LD_50_). Mice in the control group were intranasally administered 40 µL of normal saline. One hour post infection, the infected mice in each group were treated with the corresponding drugs or distilled water once a day by gavage for 5 days. On day 5 post infection, mice were euthanized by isoflurane overdose and sacrificed for lung index calculation and histopathological evaluation.

### Measurement of lung index

On the last day after the virus infection, mice in experiments 1–3 were weighed, and the lung tissues were excised from the mice and weighed immediately. Lung index was measured as described previously [32] and calculated as follows: lung index = (lung wet weight (g)/body weight (g)) × 100.

### Histopathological evaluation

Lung tissues collected in experiments 1–3 was fixed using 4% paraformaldehyde solution for more than 48 h. Following repair block, gradient alcohol dehydration, and paraffin embedding, each tissue was cut into 5-µm-thick sections and stained with hematoxylin and eosin. The pathological results were analyzed by optical microscopy. Histopathological analysis was conducted as described previously, with slight modifications [33].

### Cytokine detection

In experiment 2, the water intake, food intake, and body weight of mice were recorded daily. The right lung lobes of mice were collected to prepare 10% lung homogenates by using an electric homogenizer on ice, and the homogenates were stored at –80 °C for cytokine assays [33]. The levels of tumor necrosis factor-α (TNF-α), interleukin-2 (IL-2), IL-4, IL-5, IL-10, IL-12p70, interferon-γ (IFN-γ), and granulocyte–macrophage colony-stimulating factor (GM-CSF) in lung homogenates were detected by Luminex Liquid Chip Technology in accordance with the manufacturer’s instructions (Bio-Plex Pro™ Mouse Cytokine, Bio-Rad).

### Analysis of gut microbiome

#### Gut microbiota detection

In experiment 2, on day 4 after infection, the feces of each mouse were collected, and microbial community genomic DNA was extracted from feces samples using the E.Z.N.A.® soil DNA kit (Omega Bio-Tek, Norcross, GA) in accordance with the manufacturer’s instructions. The DNA extract was tested on 1% agarose gel, and the concentration and purity of DNA were determined by the NanoDrop 2000 UV–vis spectrophotometer (Thermo Fisher Scientific, Wilmington, NC). The hypervariable regionV3-V4 of the bacterial 16S rRNA gene was amplified with primer pairs 338F (5ʹ-ACTCCTACGGGAGGCAGCAG-3ʹ) and 806R (5ʹ-GGACTACHVGGGTWTCTAAT-3ʹ) by the GeneAmp® 9700 PCR thermocycler (Applied Biosystems, Foster City, CA), with an eight-base sequence barcode unique to each sample at the 5′-end of 338F and 806R. The PCR product was extracted on a 2% agarose gel and purified using the AxyPrep DNA gel extraction kit (Axygen Biosciences, Union City, CA) in accordance with the manufacturer’s instructions and quantified using QuantiFluor™-ST (Promega, Madison, WI). The purified amplicons were pooled and analyzed in equimolar amounts, and they were paired-end sequenced (2 × 300) on an Illumina MiSeq platform (Illumina, San Diego, CA) in accordance with the standard protocols of Majorbio Bio-Pharm Technology Co., Ltd. (Shanghai, China). The detection was performed as described previously, with slight modifications [34].

#### Analysis of gut microbiome

Alpha diversity was used to analyze the diversity and richness of microorganisms in the community, while beta diversity was used to analyze the composition of microbial communities in different samples. First, the operational taxonomic unit (OTU) information of all samples was leveled according to the lowest sequence number. On the basis of the species annotation results of all samples and the OTU abundance information, the OTU information of the same category was combined to obtain a species abundance information analysis table. The principal coordinate analysis (PCoA) method was used to analyze the difference in the gut flora between the control group and the model group, and between the model group and each drug group. Next, t-test was performed between the two groups to determine the species with significant differences at each classification level (*p* < 0.05). LEfSe (LDA effect size) analysis was performed to determine the biomarkers with a significant difference between the two groups. This analytical tool is used to discover and interpret high-dimensional biomarkers (genes, pathways, and taxa), with an emphasis on statistical significance and biological relevance and the ability to identify features of different abundances and related categories [35, 36].

### Network pharmacology analysis

The possible mechanism by which HQS exerts a therapeutic effect against influenza was analyzed by network pharmacology, as described previously, with slight modifications [37]. HQS is mainly composed of baicalein, a monomer compound of flavonoids. The actual content of baicalein is 100.6% of the labeled amount. Baicalein is considered as the only active ingredient in HQS tablets. Thus, the study of the mechanism of HQS implies the exploration of the mechanism of baicalein. Therefore, we directly analyzed here the medicinal properties and related targets and pathways of baicalein. First, the basic information about baicalein was obtained from the TCMSP platform (Chinese Medicine System Pharmacology Database and Analysis Platform) (http://tcmspw.com/index.php, Molecule ID: MOL002714). Second, the targets of baicalein were determined based on the CTD platform (http://tcmspw.com/index.php, All Interacting Gene). The chemical similarity was predicted based on the Swiss platform (http://www.swisstargetprediction.ch/index.php, Homo sapiens). The QSAR model was predicted based on the TargetNet platform (http://targetnet.scbdd.com/home/index/, Prob > 0.5). New library targets were supplemented based on the SymMap platform (https://www.symmap.org/). Third, the influenza targets and gene information were searched through the DisGeNET platform (https://www.disgenet.org/home/, Search condition, Influenza, Summary of All Gene-Disease Associations). The common targets of baicalein and influenza were screened and used to construct a protein–protein interaction (PPI) network. (STRING https://string-db.org/cgi/input.pl?sessionId=S0abM5EJpvTv&input_page_show_search = off, Organism: Homo sapiens). Fourth, by using the Omicshare platform (https://www.omicshare.com/, Homo sapiens genes), the potential target genes were subjected to Gene Ontology (GO) enrichment analysis. Fifth, all relevant significant targeting pathways were obtained by analyzing the differential genes (CTD, http://tcmspw.com/index.php, condition *P* < 0.01). The differential genes were mapped to each pathway map, and the key genes of key pathways were identified. Sixth, visual analysis was performed by Cytoscape 3.7.2 software.

### Data analysis

GraphPad Prism v.6 software was used to analyze statistical data. Data were presented as mean ± SD. For multiple groups, data were evaluated by one-way analysis of variance (ANOVA). The data of “synergistic effect” were analyzed use one-way-ANOVA followed by Tukey's HSD test. Data between two groups were statistically analyzed using Student’s t-test. A *P*-value of < 0.05 was considered significant.

## Results

### Pharmacological activity of HQS against influenza and the effect of combined application with OS

**Fig. 2:**
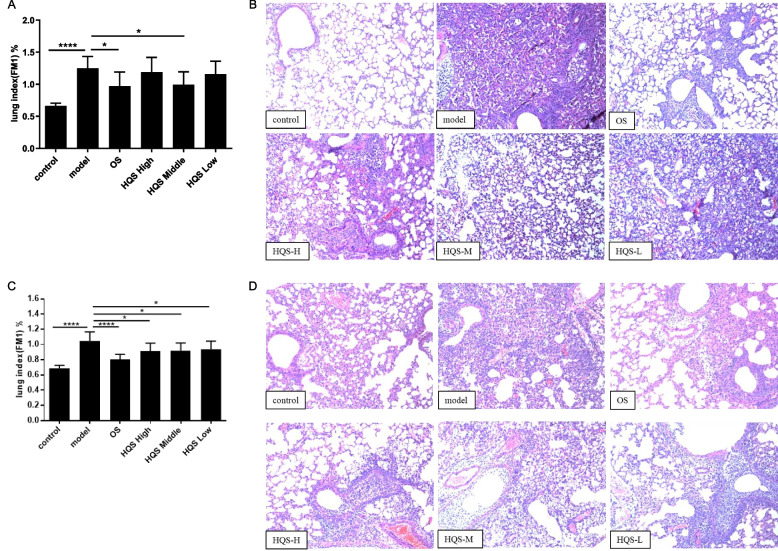
Effect of HQS and OS on mice infected with influenza. **A** (lung index, n ≥ 8) and **B** (lung pathology, × 100, *n* = 7) represent the results of experiment 1 “Therapeutic effect of HQS on influenza pneumonia in mice.” Mice were intranasally infected with 15 LD_50_ of influenza virus. Six groups of mice were simultaneously treated orally with HQS low-, middle-, and high-dose (330, 165, and 82.5 mg/kg/d), Oseltamivir (OS, 27.5 mg/kg/d), or distilled water (control and model group) for 5 days. On day 5 post infection, the lungs of the mice were collected for further analysis. **C** (lung index, *n* = 10) and **D** (lung pathology, × 100, *n* = 7) represent the results of experiment 2 “Preventive effect of HQS on influenza pneumonia in mice.” Mice were treated with HQS low-, middle-, and high-dose (330, 165, and 82.5 mg/kg/d), Oseltamivir (OS, 27.5 mg/kg/d), or distilled water (control and model group) once daily for 3 days through gavage. After the last administration, the mice were intranasally infected with 15 LD_50_ of influenza A virus. On day 4 after infection, the lungs of the mice were collected for further analysis. Results are expressed as mean ± SD, ^*^P < 0.05, ^**^P < 0.01, compared to the model group

**Fig. 3:**
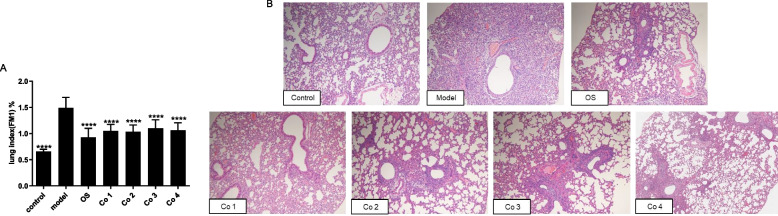
Effect of combined use of HQS and OS in experiment 3. To assess whether the use of HQS could increase the pharmacodynamic level of OS or reduce the dose of OS, 70 mice were divided into control group (distilled water); model group (distilled water); OS group (OS, 27.5 mg/kg/d); and Co 1 (HQS 165 mg/kg/d with OS 27.5 mg/kg/d), Co 2 (HQS 165 mg/kg/d with OS 14 mg/kg/d), Co 3 (HQS 165 mg/kg/d with OS 7 mg/kg/d), and Co 4 (HQS 82.5 mg/kg/d with OS 14 mg/kg/d) groups (*n* = 10). Mice were intranasally infected with 15 LD_50_ of influenza A virus. On day 5 after infection, the lungs of the mice were collected for analyzing lung index (**A**, n ≥ 9) and lung pathology (**B**, 100 × , *n* = 7). Results are represented as mean ± SD, ^*^P < 0.05, ^**^P < 0.01, compared to the model group

The effectiveness of HQS against influenza was assessed by the lung index and lung pathology. As shown in Fig. 2A and C, the lung index of mice in the model group was significantly increased as compared to that in the control group (*P* < 0.01). In contrast, 165 mg/kg HQS in experiment 1 and 330, 165, and 82.5 mg/kg/d HQS dose-dependently in experiment 2 reduced the lung index (*P* < 0.05). No apparent pathological changes were observed in the lungs of control mice (Fig. 2B and D). The model group mice showed obvious pathological changes in the lungs, such as the disappearance of alveolar structure, thickening of the alveolar septum, inflammatory cell infiltration, perivascular inflammation, and infiltration of inflammatory cells in and around the bronchial lumen. HQS (165 mg/kg/d) in experiment 1 and HQS (330 and 165 mg/kg/d) in experiment 2 improved the alveolar structure and decreased the inflammation in the lungs. Other doses of HQS were, however, less effective. OS also reduced lung index and improved lung pathology (*P* < 0.01). Overall, HQS had anti-influenza effect, and the effect of 165 mg/kg/d HQS was relatively stable. Therefore, 165 mg/kg/d HQS was chosen to determine its synergistic effects with OS. As shown in Fig. 3A, all the Co groups showed a significant decrease in the lung index as compared to the model group (*P* < 0.01); the effect was not significantly different from that of the OS group. Lung pathology was also improved in these groups as compared to that in the model group (Fig. 3B). Taken together, HQS could not improve the optimal efficacy of OS, but it could improve the efficacy of low-dose OS.

### HQS prophylactic administration improved the general state of mice infected with influenza and downregulated cytokine expression

**Fig. 4:**
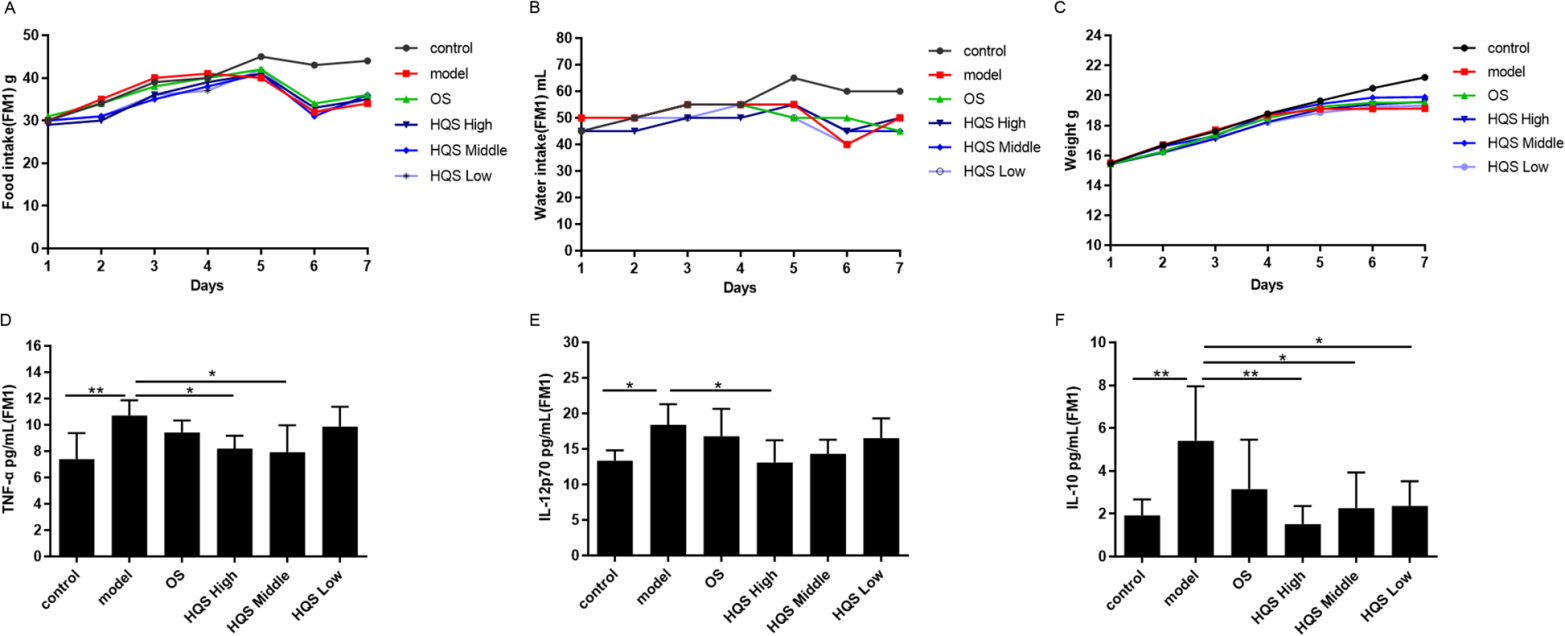
Effect of prophylactic administration of HQS and OS on the general condition and cytokine expression in mice infected with influenza. Mice were infected as described in Fig. 2. The total amount of diet (**A**), water intake (**B**), and average body weight (**C**) of mice in all groups were recorded every day (*n* = 10). On day 4 post infection, mice were euthanized, and their serum was harvested. The concentrations of the cytokines TNF-α (**D**), IL-12p70 (**E**), and IL-10 (**F**) in lung homogenates were detected by Luminex Liquid Chip Technology. Results of cytokine expression levels are represented as mean ± SD (*n* = 6), ^*^P < 0.05, ^**^P < 0.01, compared with the model group

After detecting the synergistic effect of HQS with OS, we focused on the role of HQS and the mechanism by which it prevents influenza. Before infection, the diet of mice in all HQS groups was slightly reduced. Figure 4A shows that on the 3^rd^ day (test day 6) post infection, the dietary intake of mice in all infected groups decreased sharply; however, no significant difference was observed between the groups. No significant difference was observed in water intake and body weight of mice in each group before infection (Fig. 4B and C). On the 3^rd^ day (test day 6) post infection, the water intake and body weight of mice in all groups decreased significantly. Among these groups, the model group showed the most obvious decreasing trend. The condition of mice in all administration groups was slightly better.

Next, the expression levels of the cytokines IL-12p70, TNF-α, and IL-10 were determined to evaluate the anti-inflammatory effect of HQS. In the model group, the expression levels of IL-12p70, IL-10, and TNF-α were significantly upregulated as compared to those in the control group (*P* < 0.05). The TNF-α levels in the HQS high-dose and middle-dose groups showed significant differences when compared with the model group (*P* < 0.05, Fig. 4D). The IL-12p70 expression level was significantly decreased in the HQS high-dose group (*P* < 0.05, Fig. 4E). Furthermore, the IL-10 level in all HQS dose groups was significantly reduced when compared with that in the model group (*P* < 0.05 and *P* < 0.01, respectively; Fig. 4F). For other cytokines, no significant change was observed in the model group when compared with that in the control group.

### Overall structural modulation of gut microbiota

Influenza causes significant changes in the human gut flora and triggers gastrointestinal symptoms. Considering the efficacy of HQS in preventing influenza, we hypothesized that it might also mediate changes in the intestinal flora during influenza infection. To investigate this aspect, the composition of microorganisms in mice feces on day 4 post infection was analyzed. The effective labels of all samples were clustered with 97% operational taxonomic unit (OTU). The sample size of this study was adequate, and the sequencing depth met the required standard, as shown in Supplementary Figure S1. Next, Wayne graph analysis showed that 461 OTUs were shared by 6 groups, and 40, 10, 12, 9, 3, and 26 OTUs were exclusive to the control, model, HQS_H, HQS_M, HQS_L, and OS groups, respectively (Supplementary Figure S2). Alpha diversity analysis was then performed based on the Shannon and Chao method. The results showed no significant difference between the control and model groups. The Shannon and Chao indices of the HQS_H group were significantly different from those of the model group (Supplementary Figures S3 and S4). Beta diversity analysis was performed using PCoA based on the Bray–Curtis and Euclidean algorithm at the OTU and genus levels (Supplementary Figure S5). These data analyses revealed significant differences. However, in terms of distribution, one or two cases of the crossover were observed in the groups. Samples in the control, HQS_H, and OS groups were distributed intensively and close to each other. In contrast, samples in the HQS_M and HQS_L groups were relatively dispersed and close to the control group but intersected with the model group.

**Fig. 5:**
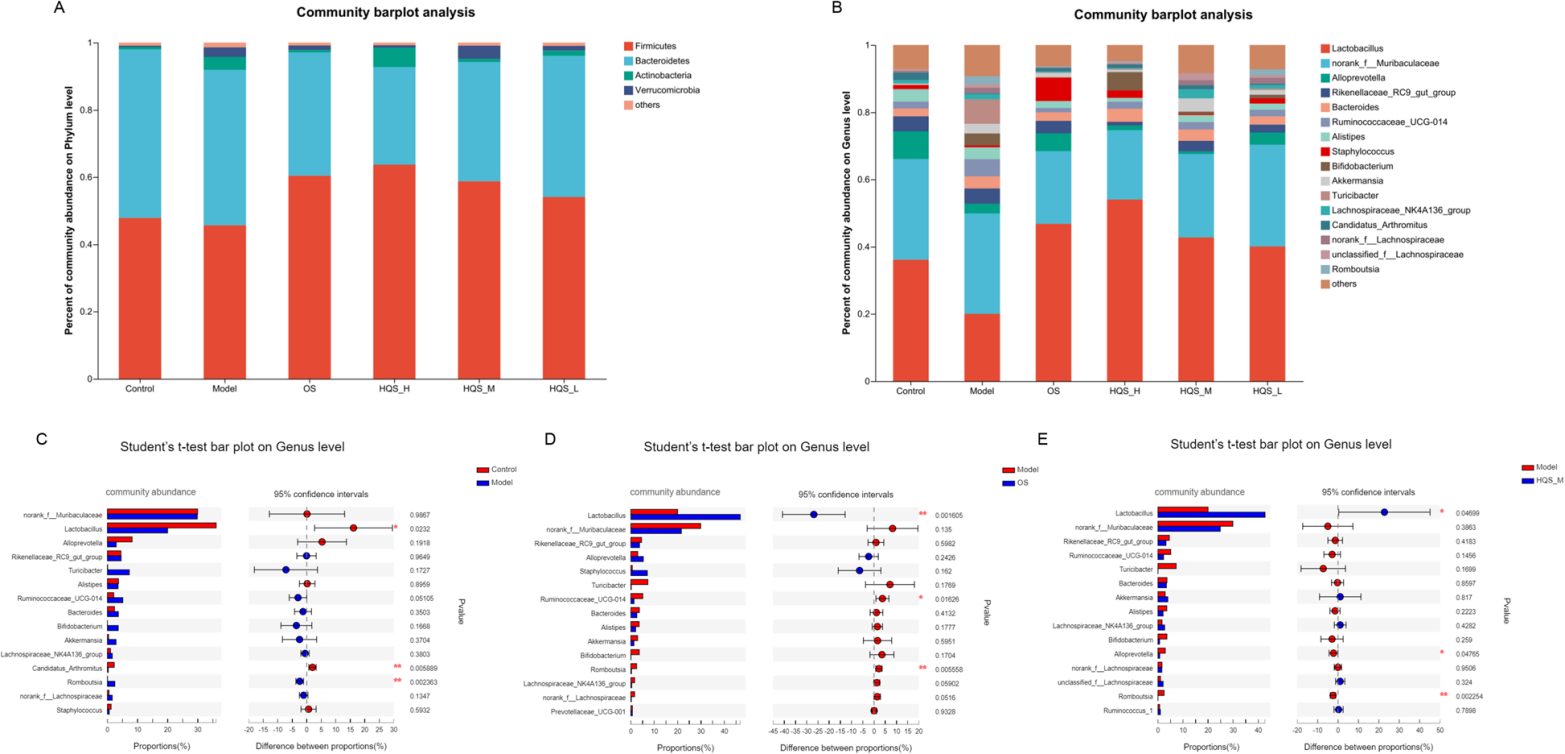
Effects of HQS on the abundance of gut microorganisms. **A** Column accumulation chart of the relative abundance of species at the phylum level (**A**) and the genus level (**B**). The abscissa represents group information, and the ordinate represents relative abundance. The sum of other species constitutes < 0.01 abundance in the sample. **C**, **D**, **E** Analysis of significant differences in community abundance between groups at the genus level. (**C**) Control vs. Model, (**D**) Model vs. OS, (**E**) Model vs. HQS_M. HQS_H, HQS_M, and HQS_L represent HQS high-, medium-, and low-dose groups, respectively. ^*^P < 0.05 and ^**^P < 0.01 indicate a significant difference between the two groups

Next, species with an abundance ratio greater than 0.01 among the groups of mice were selected on the basis of the results of species annotations, and a columnar cumulative chart of species' relative abundance was created to visually display the species with a higher relative abundance and their proportions. At the phylum level, *Firmicutes*, *Bacteroidetes*, *Actinobacteria*, and *Verrucomicrobia* were mainly the key flora. At the genus level, *Lactobacillus*, *Norank_f_Muribaculaceae*, *Alloprevotella*, *Rikenellaceae_RC9_gut_group*, and *Bacteroides* were the top five flora (Fig. 5A and B). Significant differences in the abundance of microbial communities in the different groups were also examined at the genus level (Fig. 5C, D, and E). The proportion of *Lactobacillus* was significantly decreased, and that of *Romboutsia* was markedly increased in the model group as compared to those in the control group (*P* < 0.05 and *P* < 0.01, respectively). In contrast, the abundance of this flora was markedly adjusted in the HQS_H and HQS_M groups (*P* < 0.05 and *P* < 0.01, respectively). The OS group showed the same effect (*P* < 0.01). Supplementary Figure S6 shows the analytical data of the HQS_H and HQS_L groups.

### Effects of HQS on microbiota composition in the feces

**Fig. 6:**
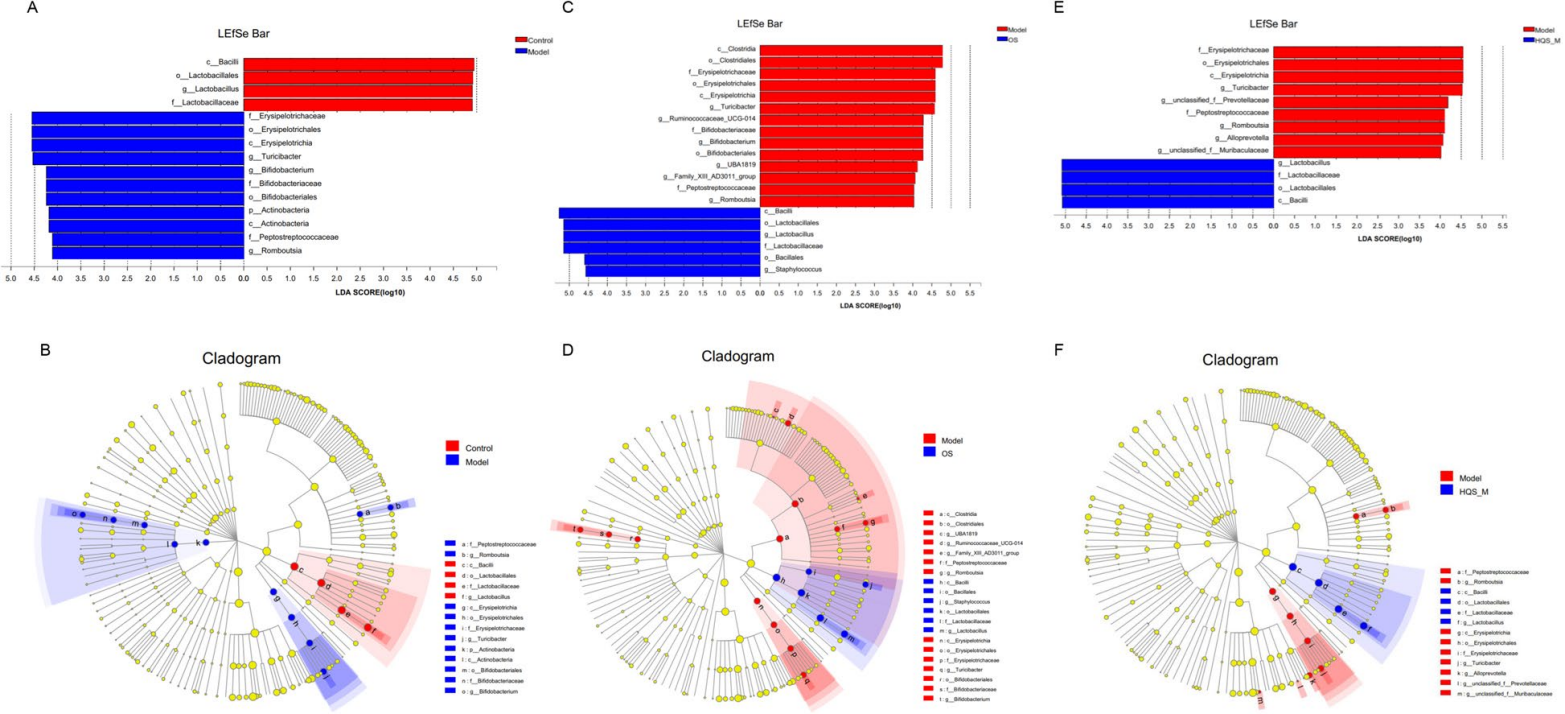
The potential gut microbial biomarkers during HQS treatment against influenza. A/C/E: The threshold on the logarithmic LDA score for discriminative features (LDA > 4.0). The higher the LDA score, the greater is the impact of species. B/D/F: Cladogram. The radiating circle from inside to outside indicates the classification level from door to genus. Each small circle at a different classification level represents the classification at that level, and the diameter of the small circle is proportional to the relative abundance of the species. Color principle: Species with no significant difference were evenly colored yellow. The red or green nodes represent the microbiome played an important role in the red or green group, respectively. A missing group indicated no significant difference in the species of this group. The name of the species in English in the picture is shown on the right. **A**, **B** Control vs. Model, **C**, **D** Model vs. OS, **E**, **F** Model vs. HQS_M. HQS_M represent HQS medium-dose group

The LEfSe method was used to identify the specific bacterial phylotypes that were differentially altered between the control, model, OS, HQS_H, HQS_M, and HQS_L groups (Fig. 6). LEfSe is a tool for discovering high-dimensional biomarkers and revealing genomic features. A cladogram and a histogram of LDA distribution were obtained from the LEfSe method, and higher LDA values (LDA > 4) were considered potential biomarkers. The results showed that the genera *c_Bacilli*, *o_Lactobacillales*, *g_Lactobacillus*, and *f_Lactobacillaceae* had significantly higher LDA values in the control group than in the model group. Moreover, *c_Bacilli*, *o_Lactobacillales*, *g_Lactobacillus*, and *f_Lactobacillaceae* were also significantly enriched in the HQS_M group. The OS group showed the same effect, but it also included *o_Bacillales* and *g_Staphylococcus*. The data of the HQS_H and HQS_L groups are shown in Supplementary Figure S7.

### Network pharmacology-based investigation of the mechanism of HQS against influenza

#### ADME-related properties of baicalein

The results of the ADME (absorption, distribution, metabolism, and excretion) analysis showed that HQS had good drug-like properties. First, the ADME-related properties of baicalein were studied through the TCMSP platform. The TCMSP database provides details regarding ADME parameters of traditional Chinese medicine components, such as oral bioavailability (OB), drug similarity (DL), Caco-2 cell permeability, and blood–brain barrier (BBB). DL is a qualitative concept used in drug design. The higher the DL, the better the pharmacokinetics and drug properties of the drug, and the set value was DL ≥ 0.18. OB is one of the most important pharmacokinetic properties of oral drugs, as it plays an important role in the efficiency of drug delivery to systemic circulation. Drugs with higher OB could achieve better curative effects while reducing drug dosage and could also reduce adverse reactions caused by the drug within a certain range. The set value was OB ≥ 30%. The molecular weight of drug-like compounds should be less than 500 g/mol. The 95% and 99% confidence ellipsoids for the BBB are considered at four prediction levels of permeability: 0 (very high), 1 (high), 2 (medium), 3 (low), and 4 (undefined). The results are shown in Table 1.

**Table 1:** Properties of baicalein

**Properties**	**Parameter**
molecular weight	270.25 g/mol
OB (%)	33.52
Caco-2	0.63
BBB level	-0.05
DL	0.21

#### Screening of common targets of HQS and influenza

Influenza-related targets were searched and filtered using the DisGeNET database, and duplicate data were removed. The keyword “baicalein” was entered into the CTD database for target search, and duplicate targets were deleted. The gene information corresponding to the target was finally obtained. Next, the above composite target dataset was imported into Cytoscape 3.7.2 software for visualization. A total of 32 targets shared by baicalein and influenza were screened (Table 2).

**Table 2:** Targets shared by baicalein and influenza

**NO.**	**Symbol**	**Description**
1	FN1	Fibronectin 1
2	PTGS2	Prostaglandin-Endoperoxide Synthase 2
3	TYR	Tyrosinase
4	MMP9	Matrix metalloproteinase 9
5	BCL2	B-cell lymphoma-2
6	CASP3	Caspase 3
7	CSF2	Colony Stimulating Factor 2
8	TNF	Tumor Necrosis Factor
9	EGFR	Epidermal growth factor receptor
10	TLR9	Toll like receptor 9
11	MAPK8	Mitogen-Activated Protein Kinase 8
12	S1PR1	Sphingosine phosphate receptor 1
13	PTGS1	Prostaglandin-Endoperoxide Synthase 1
14	NFκB1	Nuclear factor kappa-B1
15	MPO	Myeloperoxidase
16	TGFB1	Transforming growth factor β1
17	MIF	Macrophage migration inhibitory factor
18	PIK3CG	phosphoinositide-3-kinase catalytic gamma polypeptide
19	ABCB1	ATP binding cassette transporter B1
20	NOS2	Nitric Oxide Synthase 2
21	TOP1	Topoisomerase 1
22	HMOX1	Hem oxygenase1
23	TERT	Telomerase reverse transcriptase
24	MAPK1	Mitogen-Activated Protein Kinase 1
25	TTR	Transthyretin
26	JUN	Jun kinase
27	CASP8	Caspase 8
28	BACE1	β-secretase 1
29	RELA	Transcription factor p65
30	PPARG	Peroxisome Proliferator Activated Receptor Gamma
31	CFTR	Cystic fibrosis transmembrane conductance regulator
32	IL1β	Interleukin 1 Beta

### Analysis of the core target of HQS against influenza

**Fig. 7:**
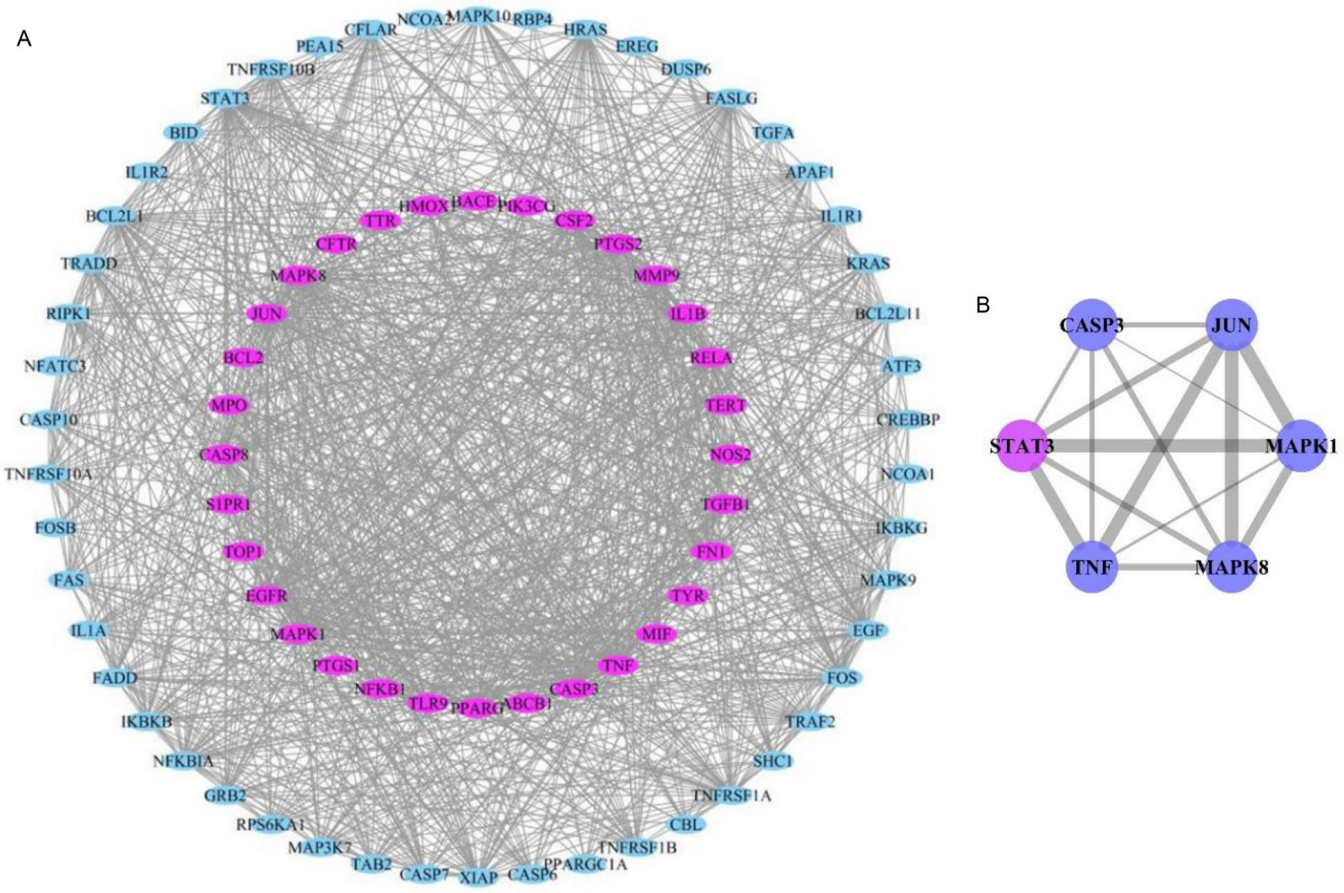
Network of the main effective targets of HQS against influenza. **A** Ovals represent the key targets. Purple ones refer to the direct effective targets, and cyan ones refer to the indirect effective targets. **B** Core targets of baicalein for preventing and curing influenza

The screened 32 target genes were uploaded to the String10.0 database (a biological database for protein–protein interaction (PPI) prediction that contains data from multiple sources such as experimental data, computational prediction methods, and public text sets) to obtain the PPI information and the data were imported into Cytoscape 3.7.2 software for visualization and PPI network construction (Fig. 7A). According to the double intermediate value of BC and DC, 6 key nodes with degree ≥ 28 were further screened, namely “core targets.” These six targets could be used as important targets of baicalein against influenza. Bar graphs of the core targets in the PPI network were drawn using GraphPad Prism 6 software (the wider the edges, the more closely related were the targets). The core targets screened included the MAPK family genes (MAPK1 and MAPK8), STAT3, CASP3, JUN, and TNF (Fig. 7B).

### Analysis of biological functions and mechanism of HQS against influenza

**Fig. 8:**
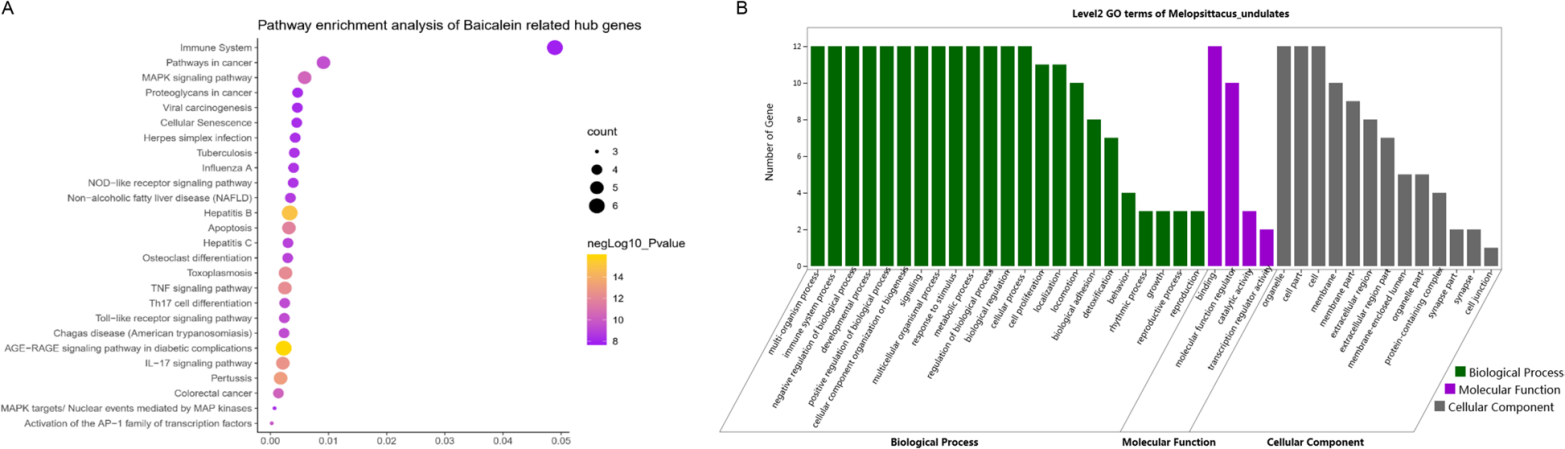
KEGG pathway and GO enrichment analysis of HQS against influenza [38–40]. **A** presents the bubble diagram of the target gene enrichment pathway of HQS. The size of the circle is proportional to the degree value. The larger the circle, the more important is the pathway in the target-path network. **B** GO analysis of baicalein targets. The abscissa represents the biological process (BP) category, molecular function (MF) category, and cell composition (CC) category associated with the targets. The ordinate represents the reaction group gene set

To demonstrate the pathway by which baicalein acts on the targets, KEGG and reactome analysis were performed. KEGG is a knowledge base for systematic analysis of gene function, and it is used to analyze the relationship between genes and biological pathways. The results with* P* < 0.01 were screened in this section, and only the top 26 pathways are shown according to the significance of the differences. The related pathways mainly included immune-related pathways, influenza A-related pathways, MAPK-related signaling pathways, TNF signaling pathways, and TLR signaling pathways (Fig. 8A) [38–40].

To illustrate the role of baicalein-related targets in gene function, Gene Ontology (GO) analysis was performed using the Omicshare database. GO analysis is widely used in the field of biology to classify gene function. This analysis includes three aspects: cellular component (CC), molecular function (MF), and biological process (BP). *P* < 0.01 is considered to have significant differences, and the results are arranged and displayed according to their degree of significance. The biological processes were mainly enriched in immune system processes and metabolic processes. The molecular functions involved in the targets were mainly enriched in drug binding, molecular function regulation, and transcriptional regulation activity. The cellular components were mainly enriched in organelles, plasma membranes, etc. (Fig. 8B).

## Discussion

The primary objective of the present study was to confirm the synergistic effect of HQS and OS on influenza and to determine the mechanism by which HQS acts against the influenza virus. Therefore, this study was divided into two parts. In the first part, we examined the efficacy level of HQS for preventing and treating influenza. In experiment 1, the role of HQS in treating influenza was investigated. In experiment 2, the role of HQS in preventing influenza was investigated. Nasal infection with the H1N1 virus led to influenza pneumonia in mice. The mice showed increased lung weight and significant pulmonary pathological changes, including inflammatory infiltration and alveolar fusion. Therefore, the lung index and lung pathological data are commonly used as basic indices to evaluate the efficacy of drugs against influenza infection. In the present study, the effect of HQS on influenza prevention and treatment was determined through the results of the lung index and lung pathology. According to the principle of the same effect, the smaller the drug dose, the better we selected the optimal effective dose of HQS and used it in combination with OS in experiment 3 to investigate the possibility of synergistic application of HQS with OS. The results revealed that HQS improved the effect of low-dose OS but could not increase the optimal effect of OS. Radix Scutellariae (RS) combined with OS inhibits the hydrolysis of OS during the absorption process and increases the absorption fraction of OS, thereby resulting in an increase in the ratio of OS concentration to OSA in plasma and urine in rats. Moreover, interestingly, the antiviral effect of OS was not affected by RS co-administration [41]. Given this context, it can be speculated that HQS has a similar effect; however, this assumption needs to be verified in further studies.

We then focused on the role of HQS in preventing influenza virus infection. The results showed that HQS dose-dependently reduced lung inflammation which confirmed the effect of baicalein on reducing inflammation caused by influenza. We further investigated the anti-influenza mechanism of HQS. On the one hand, influenza-induced infection in mice causes abnormal responses of protein pathways in tissues such as the lungs, and the reversal of abnormally altered protein pathways is confirmed to be an important mechanism through many drugs function. As reported by many studies, baicalein has multiple pharmacological effects and regulates body functions through multiple targets [42, 43]. Thus, it is crucial to investigate the targets and mechanisms of action of baicalein against influenza. Network pharmacology provides a simple platform for Chinese medicine research to evaluate the multitarget effects of drugs and to increase the understanding of the theory of biological network function [44–47]. Therefore, in the present study, a network pharmacology approach was used to comprehensively analyze and predict the targets and pathways of HQS at the protein level. Six core targets of HQS against influenza were screened by this approach, and the main pathways included the MAPK-related signaling pathway, TNF signaling pathway, TLR signaling pathway, etc.

On the other hand, the lung and intestine are closely related in response to infectious diseases [48]. Influenza infection disrupts the intestinal flora, which in turn exacerbates its severity [49]. The inhibition or modulation of the disturbed gut microbiome of influenza-infected mice by increasing beneficial flora and restoring intestinal function may inhibit or reduce the extent of influenza infection in mice. Therefore, we detected the regulatory effect of HQS treatment on the intestinal flora of H1N1 virus-infected mice. Influenza causes gastrointestinal symptoms and changes the composition of the intestinal flora [50]. The results showed that the gut microbiota composition of mice changed significantly following influenza infection. This finding suggests that gut microbiota homeostasis was disrupted in influenza mice. HQS treatment modulated the gut microbial composition of influenza-infected mice. Different doses of HQS had different degrees of inhibition of influenza infection. Similarly, the degree of modulation of the gut microbiota also depended on the HQS dose. This indicates that the effect of HQS against influenza was closely related to the regulation of gut microbiota. Furthermore, one of the most striking features of the model group mice was that the abundance of *Lactobacillus* was significantly reduced as compared to that in the control group mice. The HQS_H and HQS_M groups showed a significant increase in the abundance of *Lactobacillus*. *Lactobacillus* is an intestinal symbiotic probiotic, which plays a role in regulating the host’s immune function, thereby improving the symptoms of influenza [23, 24]. The reduced abundance of *Lactobacillus* may be an important factor in aggravating the level of influenza infection in model mice. We speculated that the increased abundance and maintenance of *Lactobacillus* in the HQS group could be beneficial for enhancing immune function, thereby enhancing protection against influenza in mice. The analysis of the interaction between *Lactobacillus* and the body will be a favorable entry point for further research on this mechanism. HQS_M also had a significant regulatory effect on these intestinal probiotics without changing the diversity of the original intestinal flora, while HQS_H showed this effect but changed the diversity of the intestinal flora. Thus, HQS_M was probably better than HQS_H. The results also showed that HQS has a similar role to OS in regulating gut microbiota. Taken together, the current study showed that HQS can modulate changes in the intestinal flora caused by influenza and can increase the abundance of probiotics; this could be an important factor for alleviating influenza infection.

## Conclusion

The present study demonstrated the efficacy of HQS against influenza and its potential as a new drug. The feasibility of using HQS in combination with OS was also analyzed. Although HQS could not improve the optimal efficacy of OS, it improved the efficacy of low-dose OS, which provides a reference for clinical application. This study also revealed a novel role of HQS in treating influenza by modulating gut microbiota. HQS enriched beneficial gut bacteria such as *Lactobacillus*. Network pharmacology studies revealed that HQS exerts anti-influenza effects through multiple targets and pathways. Thus, HQS has a high potential for treating influenza infection.

## Supplementary Information

**Additional file 1.**

## Data Availability

The datasets used in the current study are available from the corresponding author on reasonable request.

## Abbreviations

BBB: Blood–brain barrier
DL: Drug similarity
GO: Gene Ontology
HQS: Huangqin Su tablet
IL: Interleukin
OUT: Operational taxonomic unit
OB: Oral bioavailability
OS: Oseltamivir phosphate
PPI: Protein–protein interaction
TNF-α: Tumor necrosis factor-α
