# Supplementary material for: Determination of the synergistic anti-influenza effect of Huangqin Su tablet and Oseltamivir and investigation of mechanism of the tablet based on gut microbiota and network pharmacology
Source: BMC Complement Med Ther. 2023 Feb 4;23:36. doi: 10.1186/s12906-023-03858-4 (PMC9898901; doi:10.1186/s12906-023-03858-4)
Supplement: Supplementary file 1 — Additional file 1. [file 12906_2023_3858_MOESM1_ESM.docx]

**Supplementary data for ‘Determination of the synergistic anti-influenza effect of Huangqin Su tablet and Oseltamivir and investigation of mechanism of the tablet based on gut microbiota and network pharmacology’**

***Analysis on Gut Microbiome***


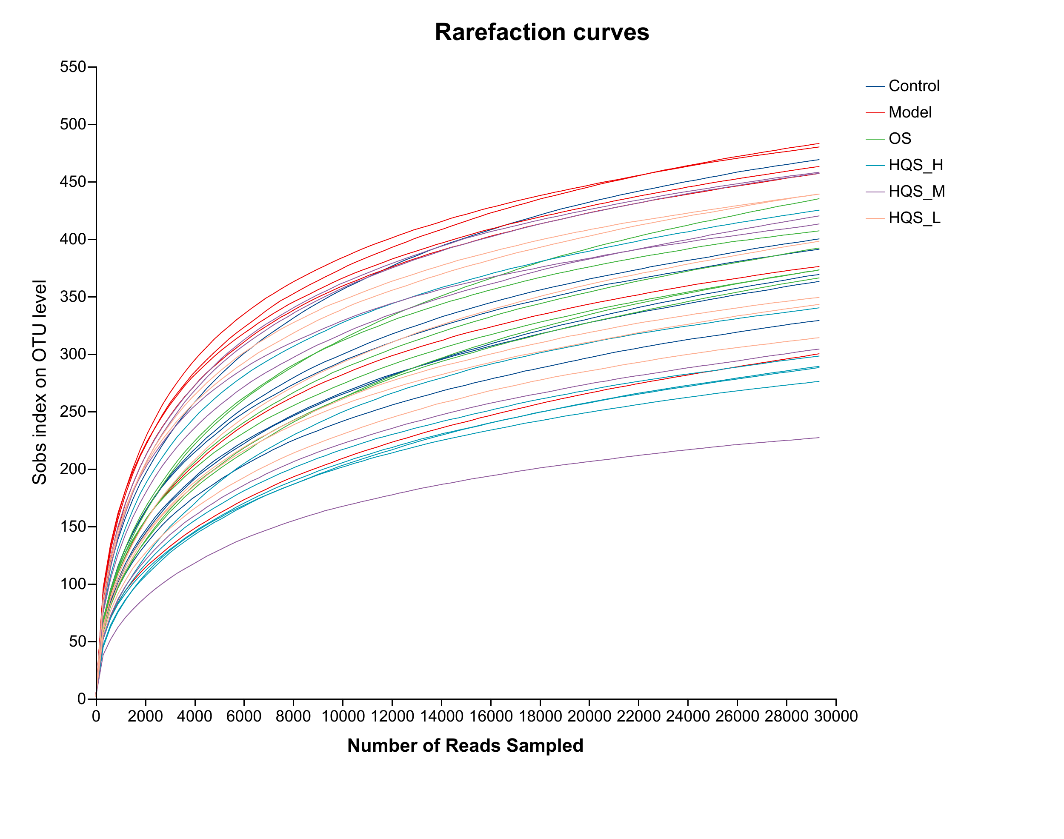


**Figure S1 Rarefaction curves.** The abscissa was the number of sequencing strips randomly selected from the sample, and the ordinate was the number of OTUs that could be constructed based on the number of sequencing strips, which was used to reflect the sequencing depth. The Rarefaction curves of each group tended to be flat, indicating that the sequencing data could reflect most of the microbial diversity information in the samples. The sample size of this study was sufficient, and the sequencing depth was up to standard.


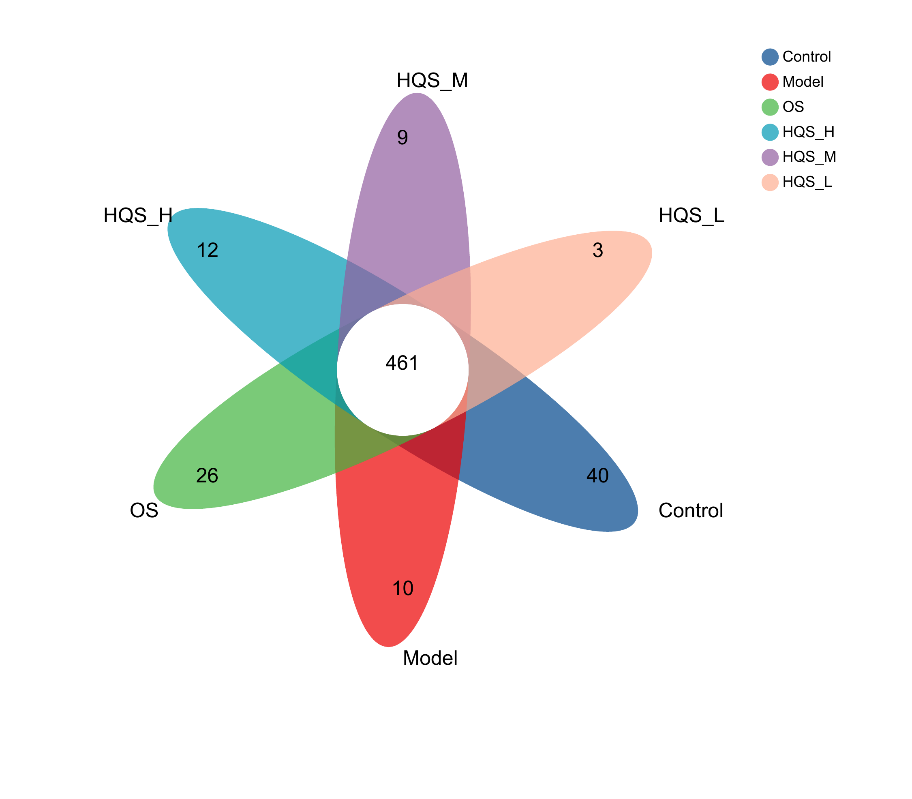


**Figure S2 Wayne graph.** Each circle in the Wayne diagram represented a group, the number of overlapping parts represented the number of OTUs shared between these groups, and the number of non-overlapping parts represented the number of unique OTUs in that group.


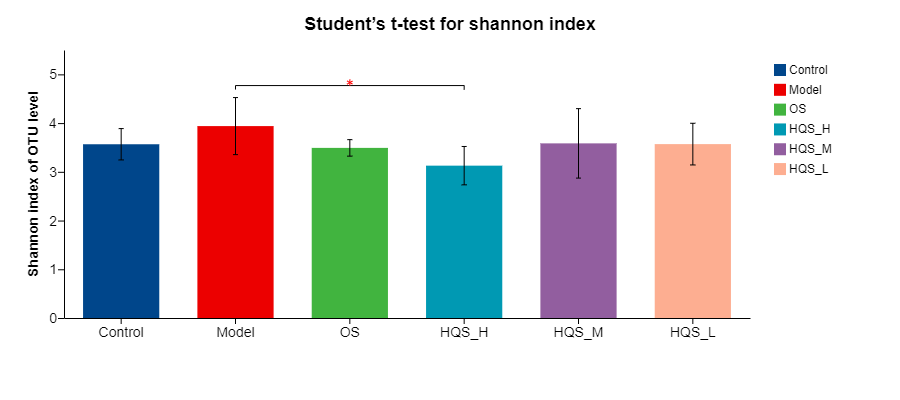


**Figure S3 Alpha diversity analysis was performed based on the Shannon method.** The larger the Shannon index, the greater the diversity of the sample.


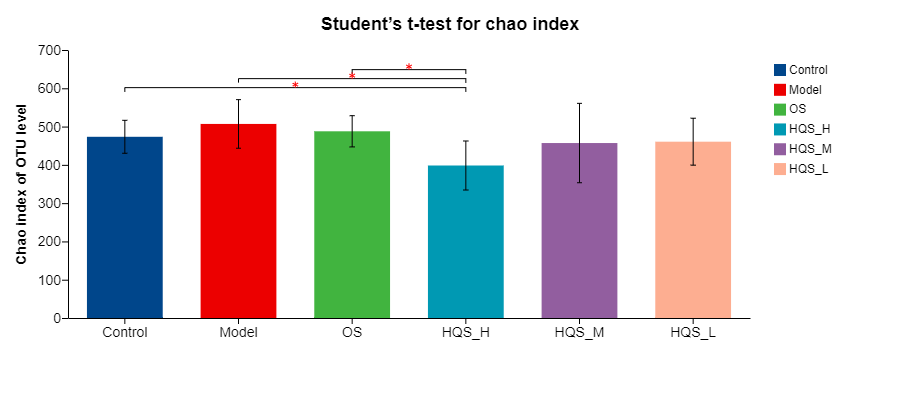


**Figure S4 Alpha diversity analysis was performed based on the Chao method.** The larger the chao index, the greater the abundance of the sample.


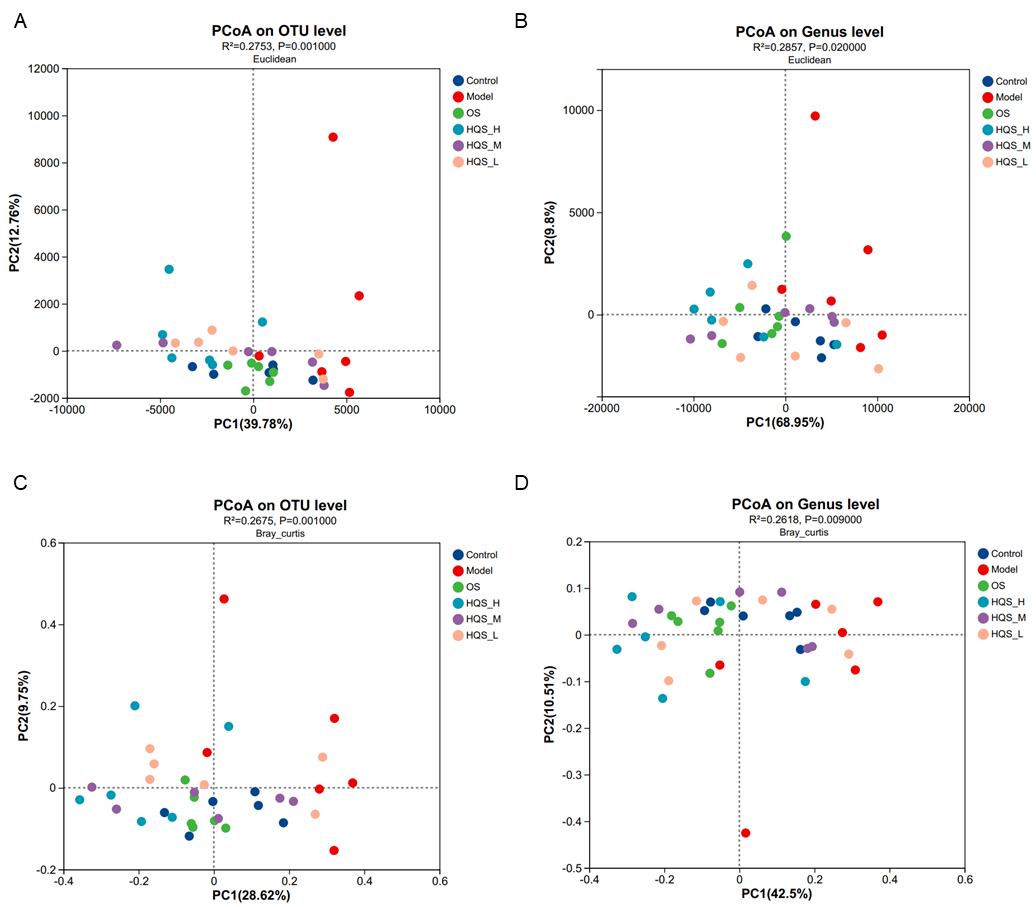


**Figure S5** **PCoA analysis.** The axis of abscissa and the axis of ordinate represent the two selected main axes, and the percentage represents the interpretation value of the main axis to the difference in sample composition. Points of different colors represent samples of different groups. The closer the points of the two samples, the more similar the species composition of the two samples. A and B are the results of analysis based on the Euclidean computational method at the OTU and genus level respectively. C and D are the results of analysis based on the Bray_curtis computational method at the OTU and genus level respectively.


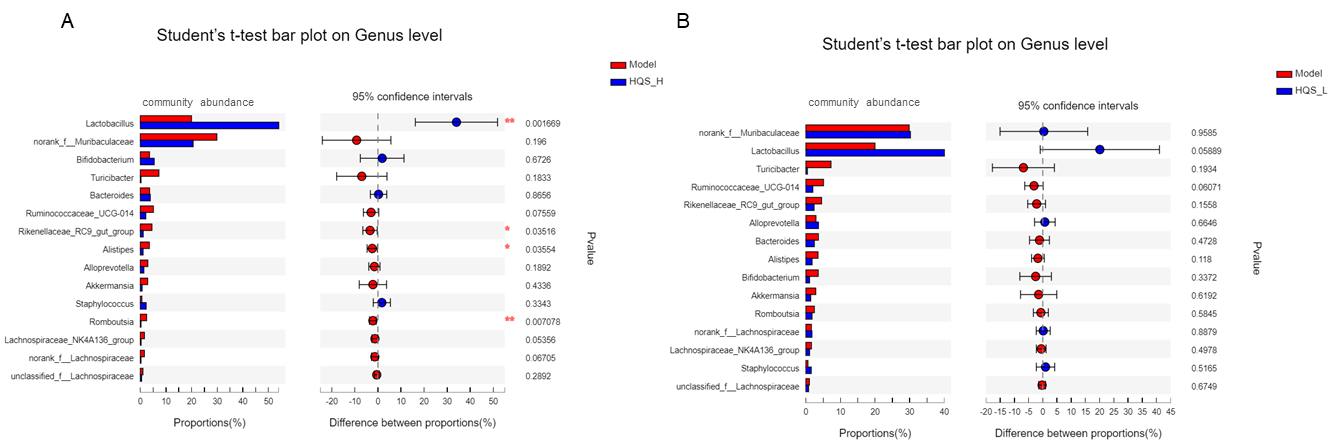


**Figure S6** Analysis of significant differences in community abundance between groups at the genus level. (A) Model vs. HQS-H, (B) Model vs. HQS-L. HQS-H and HQS-L represent HQS high- and low-dose groups, respectively. ^*^*P*<0.05 and ^**^*P*<0.01 indicate a significant difference between the two groups.


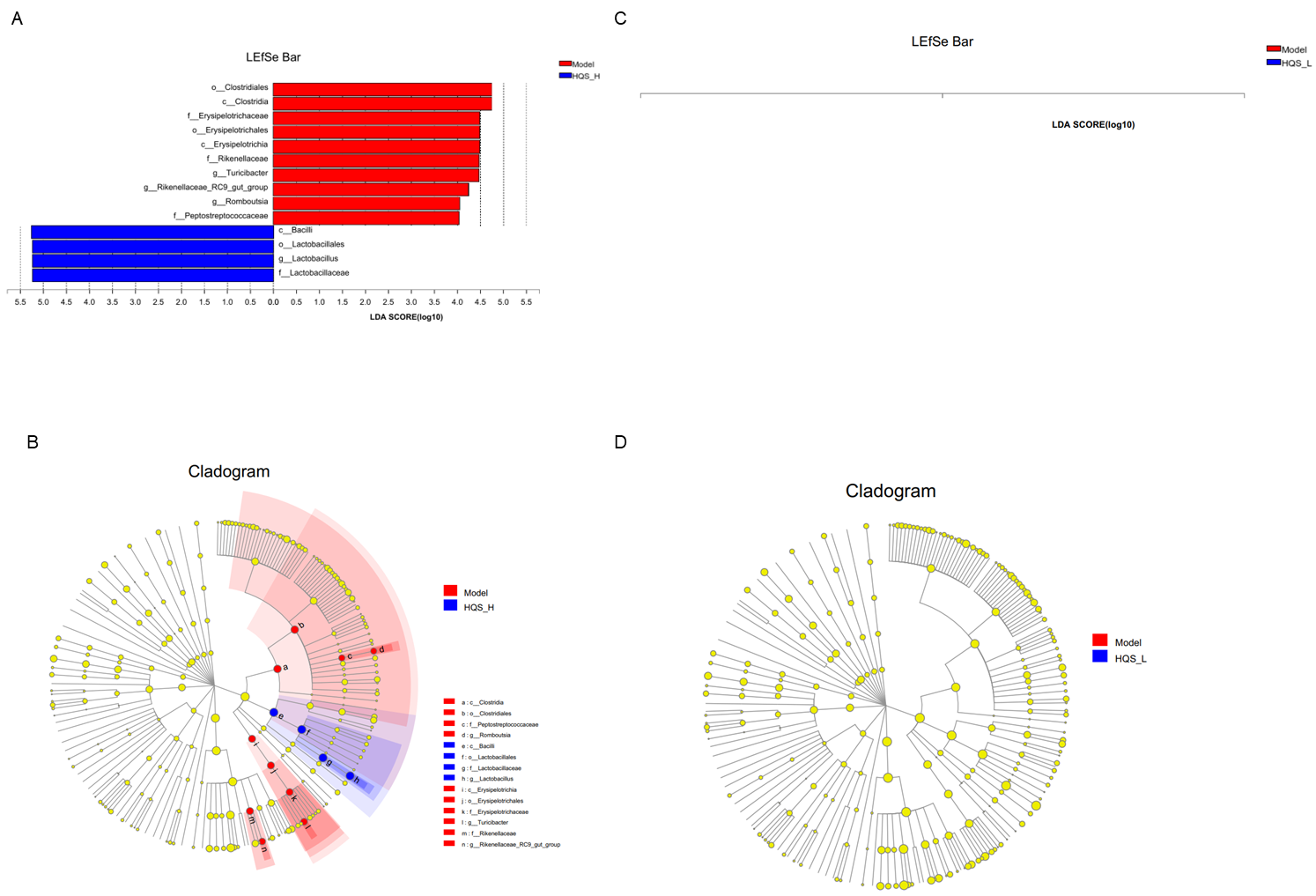


**Figure S7 The potential gut microbial biomarkers during HQS treatment against influenza.** A/C: The threshold on the logarithmic LDA score for discriminative features (LDA > 4.0). The higher the LDA score, the greater is the impact of species. B/D: Cladogram. The radiating circle from inside to outside indicates the classification level from door to genus. Each small circle at a different classification level represents the classification at that level, and the diameter of the small circle is proportional to the relative abundance of the species. Color principle: Species with no significant difference were evenly colored yellow. The red or green nodes represent the microbiome played an important role in the red or green group, respectively. A missing group indicated no significant difference in the species of this group. The name of the species in English in the picture is shown on the right. (A, B) Model vs. HQS_H, (C, D) Model vs. HQS_L. HQS_H and HQS_L represent HQS high- and low-dose groups, respectively.
